# ProToDeviseR: an automated protein topology scheme generator

**DOI:** 10.1186/s12859-025-06088-2

**Published:** 2025-03-03

**Authors:** Petar Petrov, Valerio Izzi

**Affiliations:** 1 Infotech Institute, University of Oulu, 90014 Oulu, Finland; 2 Faculty of Biochemistry and Molecular Medicine & Faculty of Medicine, Bioim Unit, University of Oulu, 90014 Oulu, Finland

**Keywords:** Protein, Topology, Sequence, Database, Scheme, Visualize

## Abstract

**Background:**

Amino acid sequence characterization is a fundamental part of virtually any protein analysis, and creating concise and clear protein topology schemes is of high importance in proteomics studies. Although numerous databases and prediction servers exist, it is challenging to incorporate data from various, and sometimes contending, resources into a publication-ready scheme.

**Results:**

Here, we present the Protein Topology Deviser R package (ProToDeviseR) for the automatic generation of protein topology schemes from database accession numbers, raw results from multiple prediction servers, or a manually prepared table of features. The application offers a graphical user interface, implemented in R Shiny, hosting an enhanced version of Pfam’s domains generator for the rendering of visually appealing schemes.

**Conclusions:**

ProToDeviseR can easily and quickly generate topology schemes by interrogating UniProt or NCBI GenPept databases and elegantly combine features from various resources.

**Supplementary Information:**

The online version contains supplementary material available at 10.1186/s12859-025-06088-2.

## Background

Proteins are complex molecules, showing an enormous structural and functional versatility [1]. Protein topology schemes are a crucial aid to virtually any research in the field of protein analysis and proteomics, as they offer a quick glance into the presence and position of structural domains, regions of functional importance, repeats, motifs, post-translational modifications (PTMs), as well as, additional sequence characteristics and peculiarities.

Protein knowledge-bases, such as UniProt [2], InterPro [3] and NCBI GenBank [4], offer summarized information on numerous protein entries. In addition, various tools for protein features prediction are available, making it possible to complement and extend the characterization of a protein by sequence analysis. Among them are the Simple Modular Architecture Research Tool (SMART) for domain identification [5] and the Eukaryotic Linear Motif (ELM) resource for short functional motif prediction [6]. Other resources are dedicated to PTMs, such as NetNGlyc [7] and NetOGlyc [8] for the detection of N/O-glycosylation, and NetPhos [9] and ScanSite [10] for phosphorylation. Additionally, intrinsically disordered (unstructured) regions of the protein, and segments within them potentially endowed with protein-binding functions, can be investigated with the IUPred/Anchor server [11]. Of the three knowledge bases, UniProt and InterPro offer graphical annotations for proteins however these, though extensive and feature-rich, are poorly suited for direct use in publications due to their spatial organization. On the other hand, feature prediction tools typically focus on simpler visual representations of the results, such as charts, and ignore topology. A notable exception here is SMART, which offers beautiful graphical annotations, albeit restricted almost exclusively to domain organization and lacking, e.g., motifs and PTMs. On the contrary, ELM is able to retrieve information from other resources and plot motif predictions along the protein context, though the verbosity and bundling of the results makes them challenging to incorporate into a publication figure. Finally, a few computational resources for the generation of custom protein schemes exist, such as MyDomains at Prosite [12] and Domain Graph [13], but they require a substantial manual work that significantly hinders their application to projects where many proteins are involved.

Here, we present the Protein Topology Deviser R package (ProToDeviseR) to produce rich, yet concise, protein schemes that are both visually appealing and publication-ready. The application can automatically retrieve information from protein databases, process raw prediction results or a user-provided table of features. ProToDeviseR then automatically transforms the information into a robust annotation code that can be rendered into a topology graph with a single click.

## Implementation

ProToDeviseR is written in R and its source code is freely available at our GitHub repository (https://github.com/izzilab/protodeviser) under GPLv3 license. The application offers a fully functional graphical user interface (GUI), implemented in R Shiny. The function that starts the GUI is called *protodeviser_ui()* and the interface has numerous dynamic tool-tips, examples, and a Help section. In addition to the R package, we also provide a server version, which requires no installation (https://matrinet.shinyapps.io/protodeviser/).

**Fig. 1:**
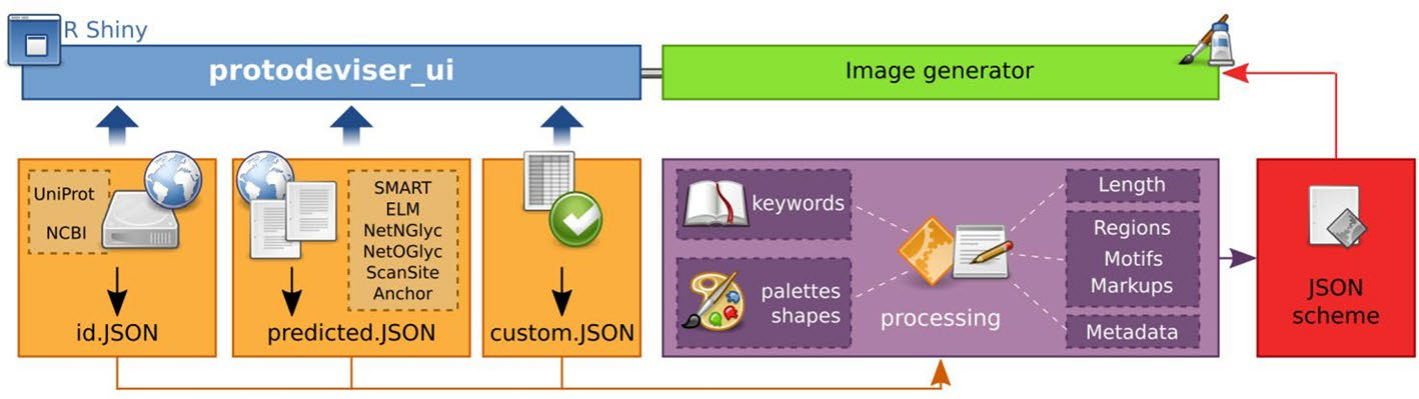
ProToDeviseR application overview. The graphical user interface *protodeviser_ui()* (blue), runs on top of the core functions *id.JSON()*, *predicted.JSON()* and *custom.JSON()* (orange), which process (purple) the input to produce a scheme in JSON code (red). Code is automatically parsed in the Image generator tab of the GUI (green)

**Fig. 2:**
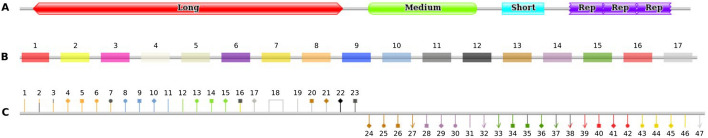
Protein features annotations. **A** Regions. Long, medium, short and three repeats are shown. At the moment, ProToDeviseR assigns regions colours anew for each protein. A rainbow gradient is used by default, where regions with the same name will have the same colour. Please note that, although coloured the same within one protein, regions may have another colour assigned to them in another protein. Shapes are: pointed: long regions (> 200 amino acids), curved: medium-sized regions (35–200 amino acids), straight: small regions (< 35 amino acids), jagged: regions designated as repeats. **B** Motifs. Signal peptide (1), coiled coil (2), low complexity (3), intrinsic disorder (4), intrinsically disordered binding (5), charged or polar amino acids patch (6), phosphorylation motif (7), glycosylation motif (8), transmembrane part (9), lipidation motif (10), cleavage motif (11), degradation motif (12), targeting motif (13), nuclear localisation or export motif (14), docking, ligand or binding motif (15), activity-related motif (16), other motif (17). **C** Markups. N-glycosylation (1), O-glycosylation (2), glycosaminoglycan (3), C-mannosylation (4), O-fucosylation (5), glycosylation unspecified (6), hydroxylation (7), prenylated (8), acylated (9), gpi (10), lipidation (11), acetylation (12), methylation (13), amidation (14), pyrrolidone carboxylic acid (15), sulfation (16), D-isomerization (17), di-sulfide bond (18), cross-linking (19), sumoylation (20), ubiquitination (21), degradation (22), cleavage (23), sorting (24), targeting (25), retaining (26), absorption (27), nuclear import (28), nuclear export (29), nuclear receptor (30), nuclear-related (31), DNA-binding (32), binding site (33), ligand binding (34), ligand site (35), docking (36), interacts with (37), flavin-binding (38), co-factor (39), active site (40), catalytic activity (41), activity regulation (42), phospho-serine (43), phospho-threonine (44), phospho-tyrosine (45), phosphorylation unspecified (46), other motif (47)

The application generates a code describing protein topology in JSON format, following the syntax used by Pfam’s [14] domain graphics tool. The four internal functions used by the GUI (Fig. 1A), namely *id.JSON()* (generates code from UniProt or NCBI GenPept ID), *predicted.JSON()* (generates code from raw results obtained from various feature prediction resources), *custom.JSON()* (generates code from a user-provided table) and *json.TABLE()* (generates a table from JSON code), can also be used independently and incorporated into other scripts, for example for batch analysis. For its annotations, ProToDeviseR searches for specific keywords (Supplementary Tables S1 and S2) and classifies protein features into three categories: Regions, Motifs and Markups. The Regions category comprises domains and other relatively long (functional) parts and repeats (Fig. 2A). The Motifs category includes short linear motifs, signal peptides, transmembrane parts, as well as disordered regions, compositional biases, coiled-coil regions, low complexity areas, etc. (Fig. 2B). The Markups category lists single site annotations, such as PTMs or sites of other importance, among which are glycosylations, phosphorylations, active sites, disulfide bonds and many more (Fig. 2C).

**Fig. 3:**
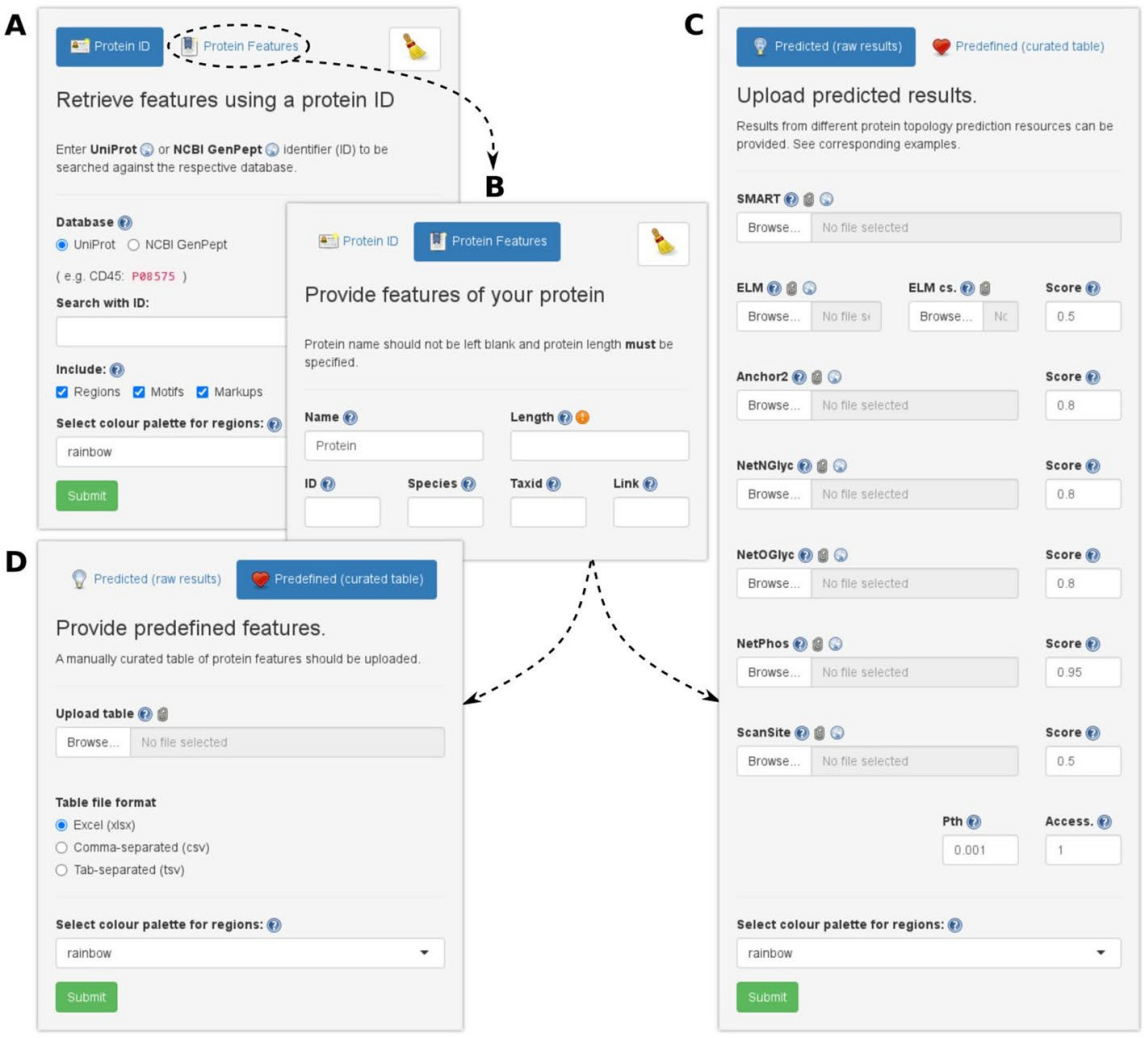
Input panel options. **A** Protein ID. Simply enter a UniProt or NCBI GenPept ID to be searched. By default, the program will search for regions, motifs and markups, but these can be deselected if desired. **B** Protein features. Enter basic metadata information about the protein—size in aa (required), name, etc. This is used for either **C** or **D**. **C** Protein features/predefined (raw results). The results from several prediction resources can be uploaded here. Threshold cut off values can be specified, where applicable. **D** Protein features/predefined (curated table). Provide a table of user-defined protein features. Accepted formats are xlsx, csv and tsv

The online and local implementations of the GUI offer identical functionality and view, with an input panel divided into two sections, “Protein ID” and “Protein features”, the latter subdivided into “Predicted” and “Predefined” (Fig. 3). The “Protein ID” section offers streamlined access to ProToDeviseR functionalities, as it simply requires a UniProt or NCBI GenPept identifier, after which all the necessary data will be automatically imported and prepared. The “Protein features” section offers a more granular approach to input protein data, with the “Predicted” tab accepting predictions from SMART, ELM, NetNGlyc, NetOGlyc, NetPhos, ScanSite and IUPred/Anchor, each settable with own cut off values, as well as fields for protein length and optional metadata, such as protein name. Alternatively, the “Predefined” tab accepts a user-prepared table of protein features as typical in data mining, when a list of manually curated features is to be visualized. Different colour palettes for domains are available at users’ preference (Supplementary Fig. S1). Upon successful integration of the input(s), the following outputs become available:

1. *Image generator*: The JSON code will appear here, ready to render the graphics upon a button click. It is important to notice that this part of the application is not directly developed by us, as it is a port of the legacy custom domain generator from Pfam. We have embedded it into ProToDeviseR for user convenience along with a few enhancements, such as proportional image size rescaling, tunable amino acid pixel size and motif opacity. In particular, adjusting the amino acid size zooms the scheme (actually, making it longer), improving its resolution, a particularly useful “trick” for short or feature-rich proteins.
2. *Table preview*: Results are shown as a table which can be inspected dynamically.

## Results

**Fig. 4:**
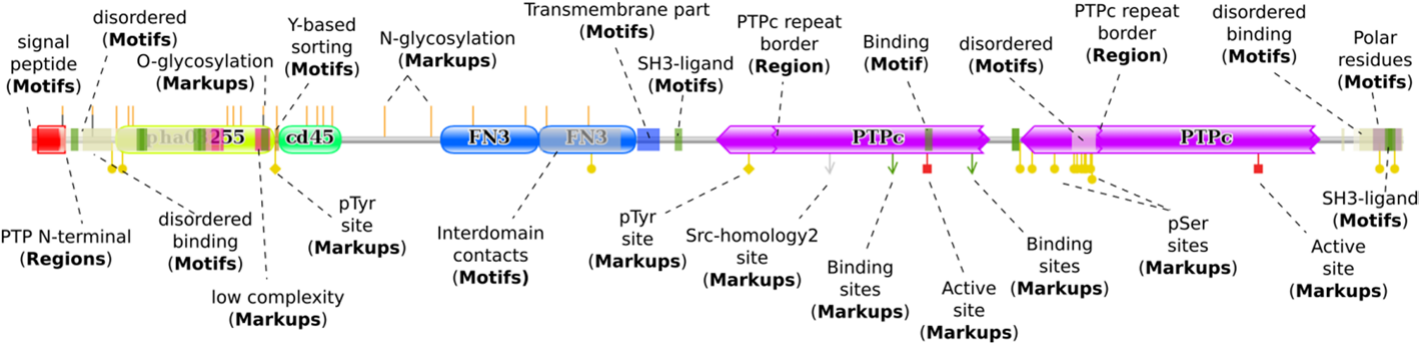
Example scheme. CD45 features were extracted from UniProt, NCBI GenPept, as well as predictions were done at SMART, ELM, NetNGlyc, NetOGlyc, NetPhos, ScanSite and IUPred/Anchor (See Fig. S2). Results were compared and a composite table was prepared and submitted to ProToDeviseR. Protein features are pointed and their respective categories are indicated in brackets

As an example, we devised topological schemes for human CD45 (Receptor-type tyrosine-protein phosphatase C), inputting either the UniProt ID P08575 or the NCBI GenPept ID NP_002829.3 into the “Protein ID” tab (Fig. 3A), as the general user would. We obtained comprehensive and mutually complementing schemes based on both resources (Supplementary Fig. S2A and B). To test for feature integration, we submitted the amino acid (aa) sequence of CD45 (1306 aa) to SMART, ELM, NetNGlyc, NetOGlyc, NetPhos, ScanSite and IUPred/Anchor, and fed the results to the “Protein features / Predicted” tab of ProToDeviseR (Fig. 3B and C), obtaining a predicted-features scheme (Supplementary Fig. S2C). Finally, to take advantage of the curated table entry functionality, we merged the results from the previous runs, removed redundant entries and submitted the combined table, following the required columns organisation (Supplementary Table S3) to the “Protein features / Predefined” tab of ProToDeviseR (Fig. 3B and D). Our example data combined the information already available at UniProt and NCBI, and added putative novel features in addition to the ones listed at the databases (Fig. 4). All the examples files are available from the Help section of the GUI.

## Conclusions

ProToDeviseR offers a fast and easy-to-use interface to comprehensively annotate proteins and devise topological schemes. It seamlessly integrates input from resources that provide only a limited visual representation of their data, or none. As a result, ProToDeviseR produces aesthetically pleasant, publication-ready graphs.

### Availability and requirements

Project name: ProToDeviseR

Project home page: https://github.com/Izzilab/protodeviser

Operating system(s): Linux, Mac, Windows, platform-independent (online version)

Programming language: R

Other requirements:

License: GPL-3.0

Any restrictions to use by non-academics: none

## Supplementary Information

Additional file 1.

## Data Availability

ProToDeviseR was developed in R v4.4.0 (https://www.r-project.org/) running on CRUX v3.7 (https://crux.nu/) distribution of GNU/Linux. All necessary software was installed from the ports freely available at the distribution’s port database (https://crux.nu/portdb/). Figures were assembled in Inkscape (https://inkscape.org/) and icons are from the Tango Desktop Project v0.8.90.

## Abbreviations

aa: Amino acid
CD: Cluster of diversification
ELM: Eukaryotic linear motif
GNU: GNU is not Unix
GUI: Graphical user interface
JSON: JavaScript object notation
NCBI: National Center for Biotechnology Information
PTM: Post-translational modification
SMART: Simple modular architecture research tool

## References

[1] Alberts B, Johnson A, Lewis J, Raff M, Roberts K, Walter P. The Shape and Structure of Proteins. In: Molecular Biology of the Cell. 4th ed. Garland Science; 2002.

[2] The UniProt Consortium. UniProt: the Universal Protein Knowledgebase in 2023. Nucl Acids Res. 2023;51:D523–31 10.1093/nar/gkac1052 PMC9825514 36408920

[3] Paysan-Lafosse T, Blum M, Chuguransky S, Grego T, Pinto BL, Salazar GA, et al. InterPro in 2022. Nucl Acids Res. 2023;51:D418–27. 36350672 10.1093/nar/gkac993 PMC9825450

[4] Clark K, Karsch-Mizrachi I, Lipman DJ, Ostell J, Sayers EW. GenBank. Nucl Acids Res. 2016;44:D67-72. 26590407 10.1093/nar/gkv1276 PMC4702903

[5] Letunic I, Khedkar S, Bork P. SMART: recent updates, new developments and status in 2020. Nucl Acids Res. 2021;49:D458–60. 33104802 10.1093/nar/gkaa937 PMC7778883

[6] Kumar M, Michael S, Alvarado-Valverde J, Mészáros B, Sámano-Sánchez H, Zeke A, et al. The eukaryotic linear motif resource: 2022 release. Nucl Acids Res. 2022;50:D497-508. 34718738 10.1093/nar/gkab975 PMC8728146

[7] Gupta R, Brunak S. Prediction of glycosylation across the human proteome and the correlation to protein function. Pac Symp Biocomput Pac Symp Biocomput. 2002;310–22. 11928486

[8] Steentoft C, Vakhrushev SY, Joshi HJ, Kong Y, Vester-Christensen MB, Schjoldager KT-BG, et al. Precision mapping of the human O-GalNAc glycoproteome through simplecell technology. EMBO J. 2013;32:1478–88. 23584533 10.1038/emboj.2013.79 PMC3655468

[9] Blom N, Sicheritz-Pontén T, Gupta R, Gammeltoft S, Brunak S. Prediction of post-translational glycosylation and phosphorylation of proteins from the amino acid sequence. Proteomics. 2004;4:1633–49. 15174133 10.1002/pmic.200300771

[10] Obenauer JC, Cantley LC, Yaffe MB. Scansite 2.0: Proteome-wide prediction of cell signaling interactions using short sequence motifs. Nucl Acids Res. 2003;31:3635–41. 12824383 10.1093/nar/gkg584 PMC168990

[11] Erdős G, Pajkos M, Dosztányi Z. IUPred3: prediction of protein disorder enhanced with unambiguous experimental annotation and visualization of evolutionary conservation. Nucl Acids Res. 2021;49:W297-303. 34048569 10.1093/nar/gkab408 PMC8262696

[12] Sigrist CJA, de Castro E, Cerutti L, Cuche BA, Hulo N, Bridge A, et al. New and continuing developments at PROSITE. Nucl Acids Res. 2013;41:D344–7. 23161676 10.1093/nar/gks1067 PMC3531220

[13] Ren J, Wen L, Gao X, Jin C, Xue Y, Yao X. DOG 1.0: illustrator of protein domain structures. Cell Res. 2009;19:271–3. 19153597 10.1038/cr.2009.6

[14] Mistry J, Chuguransky S, Williams L, Qureshi M, Salazar GA, Sonnhammer ELL, et al. Pfam: the protein families database in 2021. Nucl Acids Res. 2021;49:D412–9. 33125078 10.1093/nar/gkaa913 PMC7779014

