# Supplementary material for: ProToDeviseR: an automated protein topology scheme generator
Source: BMC Bioinformatics. 2025 Mar 3;26:71. doi: 10.1186/s12859-025-06088-2 (PMC11874827; doi:10.1186/s12859-025-06088-2)
Supplement: Supplementary file 1 — Additional file 1. [file 12859_2025_6088_MOESM1_ESM.docx]

**ProToDeviseR: an automated protein topology scheme generator**

Petar Petrov^1,*^ and Valerio Izzi^1,2^

^1^ Infotech Institute, University of Oulu, FI-90014 Oulu, Finland.

^2^ Faculty of Biochemistry and Molecular Medicine & Faculty of Medicine, BioIM Unit, University of Oulu, FI-90014 Oulu, Finland.

^*^To whom correspondence should be addressed.

**Supplementary information**


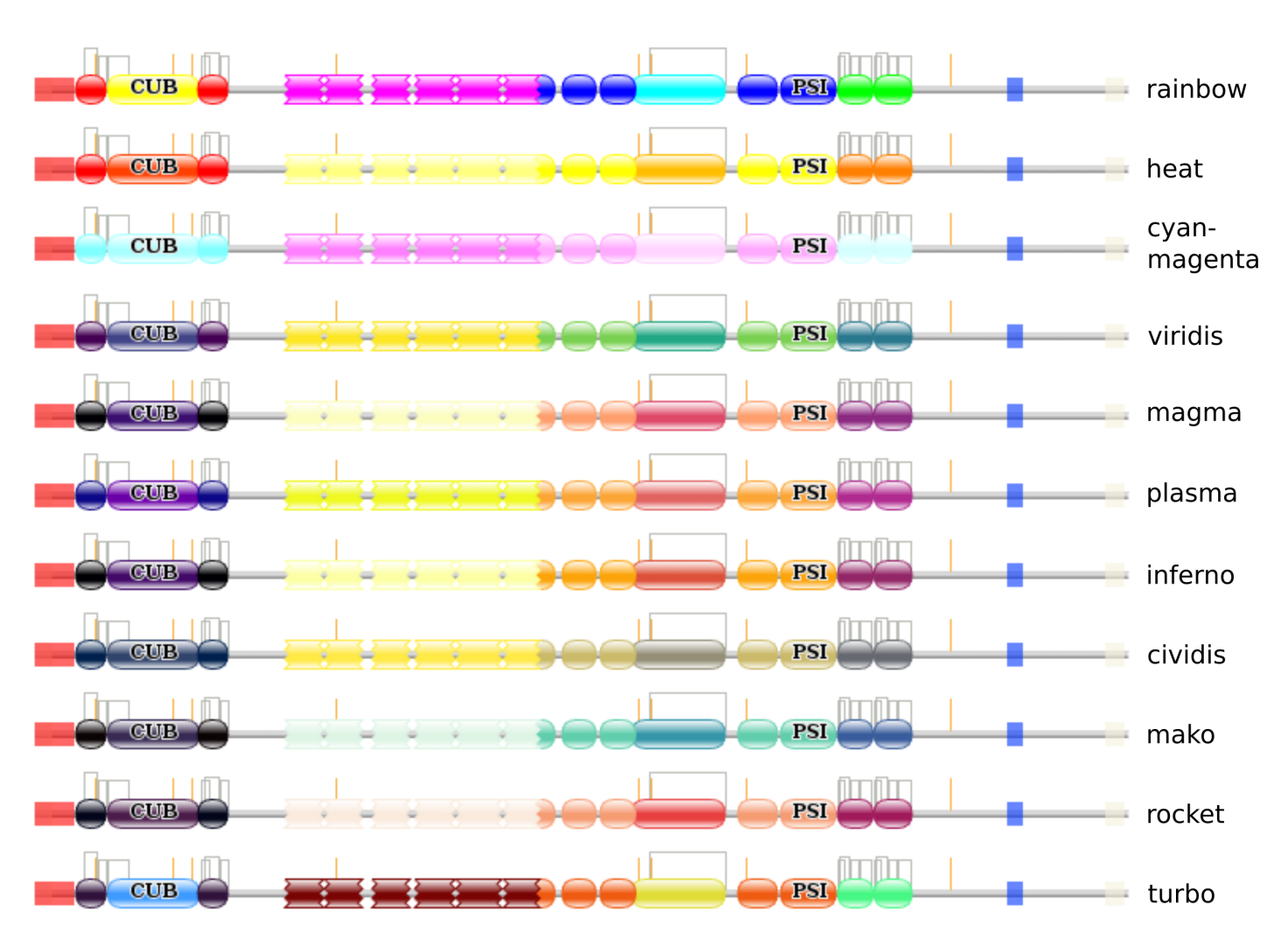


**Figure S1. Colour palettes for regions (domains) provided by ProToDeviseR.** Currently, 11 palettes are offered. As a demonstration, the multi-domain Human Attractin-like protein 1 (ATRN1_HUMAN of 1379 (aa), UniProt ID: Q5VV63) was analysed, using each gradient.


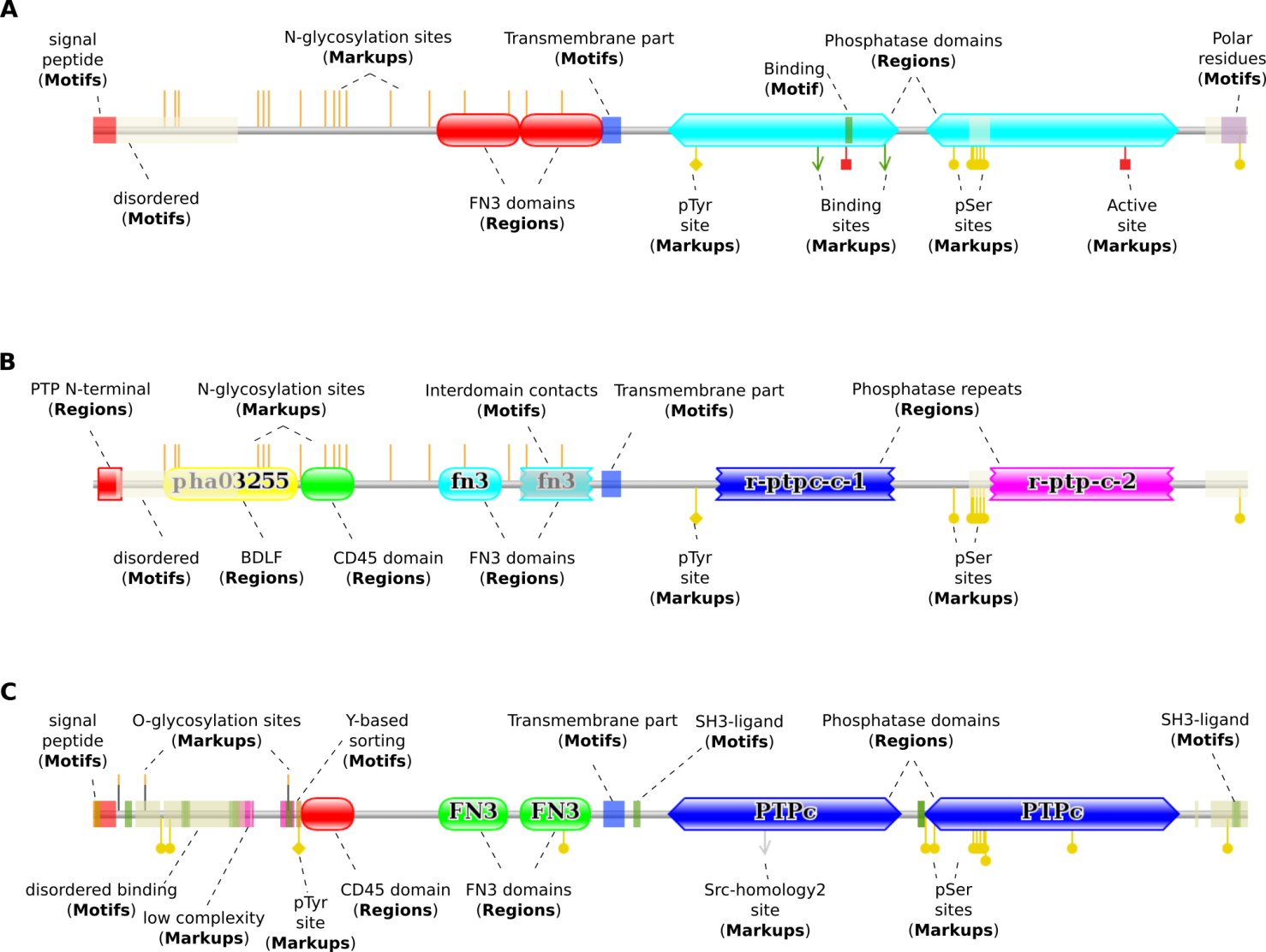


**Figure S2. Schemes of CD45 produced by different search strategies of ProToDeviseR.** A) The “Protein ID” tab was used with ID “P08575” to retrieve information about the protein from UniProt. B) The “Protein ID” tab was used with ID “NP_002829.3”, analogously for NCBI GenPept. The database denotes the second FN3 domain and the phosphatase domains as repeats, hence the jagged edges. C) Predicted features for sequence with Uniprot ID “P08575”, were submitted at the “Protein features / Predicted” tab. Cut off values were as follows: SMART (NA), ELM (Score > 0, no filtering applied in order to show all motifs), NetNglyc (Score > 0.95), NetOGlyc (Score > 0.95), NetPhos (Score > 0.99), ScanSite (Score > 0.5, Percentile threshold > 0.001, Accessibility > 1) and IUPred3/Anchor2 (Score > 0.6).

| **Motif class** | **Keywords** |
| --- | --- |
| activity_regulation | co-factor, active site, catalytic activity, activity regulation, activity |
| charged_polar_reg | basic, acidic, charged, polar |
| cleavage | cleave, cleavage |
| coiled_coil | coiled coil |
| degradation | degrad, degron, destruct |
| disordered_region | disorder |
| disor_region_bind | disorder bind |
| docking_ligand | ligand, bind, docking, interact |
| glycosylation | glycosaminoglycan, mucopolysaccharide, O-fucos, C-mannos, N-glyc, O-glyc, glycosylation |
| lipidation | prenyl, isopren, farnes, geranyl, dolichol, caax, acylat, myrist, palmit, gpi, glycosylphosphatidylinositol, phosphoethanolamine, lipid |
| low_complexity | low complex |
| nuclear_related | nucleus, nuclear import/localization, nuclear export/localization, zink finger, dna |
| phosphorylation | ser, thr, tyr, phospho, kinase |
| signal_peptide | signal, signal peptide |
| targeting | sumo, absorb, absorption sort, target |
| transmembrane | trans-membrane |

**Table S1.** Columns for a user-prepared table with protein topology annotations.

| **Markup class** | **Keywords** |
| --- | --- |
| absorption | absorption, absorb |
| acetylation | acetyl |
| active_site | activ site |
| activity_regulation | activ regulat |
| acylated | acylat, myrist, palmit |
| amidation | amide, amidation |
| binding_site | bind |
| catalytic_activity | catal activ |
| cleavage | cleave, cleavage |
| C_mannosylation | C-mannos |
| cofactor | co-factor |
| cross_link | cross-link |
| degradation | degrad, destruct, degron |
| diSulfide_bridge | disulf |
| dna_binding | dna bind |
| docking | docking |
| flavin_binding | flavin, flavo, fmn, fad |
| glycosaminoglycan | glycosaminoglycan |
| glycosylation | glycosylation, mucopolysaccharide |
| gpi | gpi, glycosylphosphatidylinositol, phosphoethanolamine |
| hydroxylation | hydroxylation, hydrocxy proline/lysine/phenylalanine/arginine/asparagine/aspartate |
| interacts_with | interact |
| isomerization | isomerization, D- |
| ligand_binding | ligand bind |
| ligand_site | ligand |
| lipidation | lipid |
| methylation | methyl |
| N_glycosylation | N-glyc |
| nuclear_export | nucleus/nuclear export |
| nuclear_import | nucleus/nuclear import/localization |
| nuclear_receptor | nucleus/nuclear receptor |
| nuclear_related | nucleus, nuclear |
| O_fucosylation | O-fucos |
| O_glycosylation | O-glyc, O-GalNAc, O-mucin |
| phosphorylation | phosphorylation |
| PhosphoSerine | Ser-phospho, Ser-kinase |
| PhosphoThreonine | Thr-phospho, Thr-kinase |
| PhosphoTyrosine | Tyr-phospho, Tyr-kinase |
| prenylated | prenyl, isopren, farnes, geranyl, dolichol, caax |
| pyrrolidone | pyrrolidone, pyroglutamic, pyroglutamate |
| retaining | retain |
| sorting | sort |
| sulfation | sulfation, sulphotyr, sulphothr, sulphoser |
| sumo | sumo |
| targeting | target |
| ubiquitin | ubiquitin |

**Table S2.** Keywords used to classify markups.

| **Column name** | **Description** |
| --- | --- |
| type | classification of the feature (string). Accepted are: regions, motifs or markups. |
| start | start coordinate (numeric) |
| end | stop/end coordinate (numeric). If you denote a markup site, use the same coordinate for start and end. |
| text | short name of feature (string). |
| description | longer description of feature (string). |
| scoreName | if feature was predicted, name of the prediction score (string). Optional |
| score | actual score value (numeric). Optional |
| database | source of feature information (string). Optional |
| accession | database identifier of feature (string). Optional |
| sequence | actual sequence of feature, as amino acids or regex (string). Optional |
| target | if a feature interacts with another partner, indicate partner (string). Optional |

**Table** S3. Columns for a user-prepared table with protein topology annotations.
